# Micromagnetic and Microstructural Characterization of Ferromagnetic Steels in Different Heat Treatment Conditions

**DOI:** 10.3390/s22124428

**Published:** 2022-06-11

**Authors:** Werner Ankener, David Böttger, Marek Smaga, Yasmine Gabi, Benjamin Strass, Bernd Wolter, Tilmann Beck

**Affiliations:** 1 Institute of Materials Science and Engineering, TU Kaiserslautern, 67663 Kaiserslautern, Germany; ankener@mv.uni-kl.de (W.A.); beck@mv.uni-kl.de (T.B.); 2 Fraunhofer-Institute for Nondestructive Testing (IZFP), Campus E31, 66123 Saarbrücken, Germany; david.boettger@izfp.fraunhofer.de (D.B.); yasmine.gabi@izfp.fraunhofer.de (Y.G.); benjamin.strass@izfp.fraunhofer.de (B.S.); bernd.wolter@izfp.fraunhofer.de (B.W.)

**Keywords:** microstructure, hardness, X-ray diffraction, magneto-mechanical correlation, 3MA

## Abstract

The paper addresses the investigation of microstructures from AISI 52100 and AISI 4140 in hardened as well as in quenched and tempered conditions. The specimens are compared in terms of their magnetic hysteresis and their microstructural and mechanical properties. Material properties were determined by hardness, microhardness, and X-ray diffraction measurements. Two different approaches were used to characterize magnetic properties via a hysteresis frame device, aiming, on the one hand, to record the magnetic hysteresis with established proceedings by setting a constant magnetic flux and, on the other hand, by offsetting a constant field strength to facilitate reproducibility of the results with other micromagnetic measurement systems. Comparable differences in both the micromagnetic and the mechanical material properties could be determined and quantified for the specifically manufactured specimens. The sensitivity of the magnetic hysteresis and, determined from that, the relationship between magnetic flux and magnetic field strength were confirmed. It was shown that a consistent change in hysteresis shape from hardened to high temperature tempered material states develops and that this change allows the characterization of different materials without the need to adjust magnetization parameters. Repeatedly, an increase in remanence with decreasing hardness was found for both test approaches. Likewise, a decreasing coercivity and increasing maximum magnetic flux could be detected with decreasing retained austenite content. The investigated correlations should thus contribute to the calibration of comparable measurement systems through the holistic characterized specimens.

## 1. Introduction

For quantification and comparability of mechanical material properties measured by means of micromagnetic NDT (non-destructive testing) systems, reliable calibration of the measuring system on selected material conditions is mandatory. The use of micromagnetic NDT in manufacturing and quality control is well-established for the efficient assessment of specific material properties [1,2]. Micromagnetic techniques offer great potential for the characterization of mechanical and microstructural properties of ferromagnetic material states [3,4,5,6,7]. Furthermore, due to the non-destructive and, in many cases, contactless measuring technique, micromagnetic NDT offers excellent opportunities for in-process measurements for process monitoring during transient machining processes of ferromagnetic materials [8]. This results in new possibilities for indirect identification of selected process and component conditions that cannot be detected with conventional measurement technology due to the measurement restrictions during the process (in-process). In the following, indirect measurement of mechanical or microstructural material properties by micromagnetic NDT means the quantitative determination of such properties based on models correlating the non-destructively measured electromagnetic material properties (micromagnetic parameters) of the component and its mechanical or microstructural material properties [8]. These methods and, especially their combination, are well established in the field of NDT. Several micromagnetic methods exist, e.g., Barkhausen noise, harmonic analysis of the tangential magnetic field strength, multi-frequency eddy current analysis, and incremental permeability. These methods are often methodically and technically combined like in 3MA. Micromagnetic methods allow non-destructive characterization of all ferromagnetic materials and reveal magnetic interactions with the microstructure and stresses at the microscopic level. The analysis depth of the micromagnetic applications is limited due to the skin effect. This maximum electromagnetic interaction depth depends on the design of the measurement equipment and the applied magnetic field strength and excitation frequency. In addition, the material properties (conductivity and magnetic hysteresis), as well as the size and shape of the investigated specimen, are influential. Determination of material damage and degradation, hardening depth, residual stress profiles, steel grade classification, or coating thicknesses are just a few possible applications [9]. The inaccuracy and reliability of these techniques depend to a large extent on the calibration of the micromagnetic NDT system and on the quality of the described underlying correlations [10]. The development of robust sensors, the definition of significant signal features, and the implementation of powerful machine learning algorithms to ensure reliable quantitative target prediction based on measurement parameters pose challenges for the industrial applicability of micromagnetic NDT systems [11]. For a better understanding of these relations, especially regarding the sensitivity of micromagnetic material properties to selected heat treatment conditions of different materials, it is necessary to characterize specifically manufactured steel specimens in terms of their micromagnetic and mechanical properties as well as by X-ray diffraction.

The aim of this study is thus a detailed comparison of the mechanical and microstructural material properties and the micromagnetic properties. For this purpose, specimens from two low-alloy steel materials in different heat treatment conditions have been investigated. The materials—AISI 52100 and AISI 4140—were used in hardened, and in quenched and tempered conditions, respectively because, on the one hand, they are widely used in industrial applications such as rolling bearings and automotive steering components after their final machining [7,12], and, on the other hand, these steel grades have a broad variety of mechanical properties, which are decisive for industrial application, and can be realized by heat treatment. By selecting the appropriate heat treatment parameters, quenching and tempering offer the possibility of adapting the hardness and the retained austenite (RA) content of the material to different requirements before machining [13]. To robustly detect these material properties, which are often affected by batch deviations, it is necessary to accurately correlate the measured micromagnetic parameters with the target quantities. This comparison should enable further quantitative correlations between these quantities and thus contribute to the calibration of micromagnetic NDT systems. In particular, the sensitivity of the magnetic measurement quantities to microstructural and mechanical material differences should be described. The focus here is on hardness and the RA content determined by X-ray since these material properties have a direct effect on machinability and service properties such as wear-resistance and fatigue life [14].

The influence of selected microstructural properties on individual micromagnetic parameters has been described in several publications [15,16,17]. The resulting conclusions focus on a specific material or material state. To comprehensively present the relationships between micromagnetic, microstructural, and mechanical parameters with a uniform test arrangement, these are examined below for different materials and material states. Another challenge that needs to be solved under practical conditions is to resolve the superposition of the influencing factors mentioned, which lead to ambiguities in the magnetic hysteresis parameters (e.g., coercive field strength H_C_ and remanence Br). This separation of the different influencing factors can be achieved based on the measurement of the full magnetic hysteresis and its characteristic values of specifically manufactured specimens that are introduced in the following.

## 2. Materials and Methods

**Figure 1:**
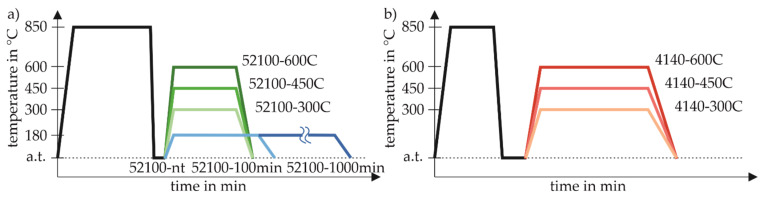
Schematic illustration of the different heat treatments applied to the specimens. (**a**) The heat treatments temperature-time curves applied on the AISI 52100 batch, (**b**) applied on the AISI 4140 batch.

**Figure 2:**
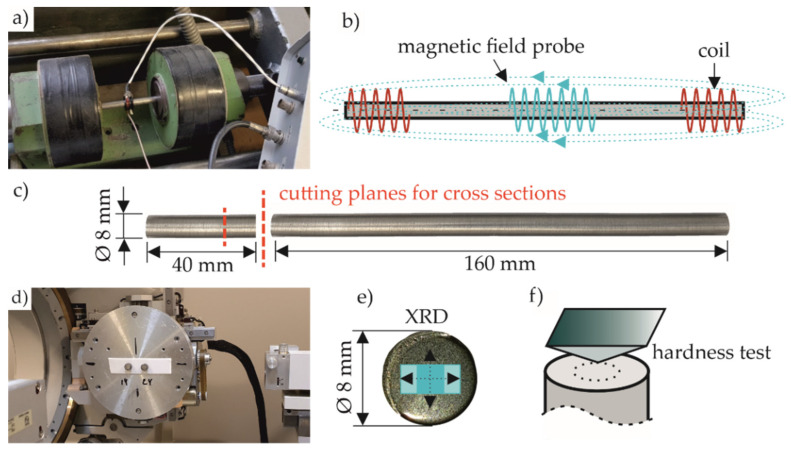
(**a**) Hysteresis frame device and (**b**) schematic illustration of the respective magnetic field in the sample, (**c**) specimen geometry for microstructural investigations and magnetic testing, (**d**) specimens attached to the XRD goniometer, (**e**) cross-section geometry showing the actual XRD spot sizes for phase analysis and the two directions of residual stress measurements, and (**f**) measuring positions of hardness tests.

Cylindrical specimens turned from billet material were used with an initial diameter of 40 mm (ASISI 52100) and 20 mm (AISI 4140), respectively. Both types of specimens were machined into cylindrical slabs of 200 mm length and 8 mm diameter. The batch-specific chemical compositions of the specimens were determined by optical emission spectroscopy and are given in Table 1. These bars were separated before heat treatment (cf. Figure 1) according to the geometry shown in Figure 2c). This provides the best possible microstructural comparability between the 40 mm and 160 mm long segments.

All investigated specimens were subsequently heat-treated in an upright position in specially prepared retaining racks. This ensured that a homogeneous heat distribution was achieved across all specimens. The parameters of the heat treatment aimed at achieving homogeneously distributed but, between the batches, clearly distinguishable properties over the entire sample cross-section, which was achieved primarily through the different tempering processes (Table 2). For this purpose, all specimens of the respective batch were austenitized together in a vacuum furnace and quenched by means of helium gas. The cooling rates achieved in this process are comparable to those of conventional oil quenching, but allow the quenching to be as uniform as possible [18,19]. The temperature-time curves of all heat treatments are shown schematically in Figure 1, illustrating the division into three different tempering temperatures per material and, additionally, the variation of the tempering time at low tempering temperatures of the AISI 52100 batch.

The target heat treatment states of the specimens can be roughly divided into three sets, indicated by the colors blue, green, and red as shown in Figure 1. This color scheme represents the following sets of varied tempering times of AISI 52100 in blue, the tempering temperature of AISI 52100 in green, and the tempering temperature of AISI 4140 in red. Large microstructural differences are to be expected between these sets, which allows for a sufficiently broad variation of specimen properties. Within the respective sets, the focus of the investigations is on the sensitivity of the measuring systems used regarding the specifically varied heat treatment parameters.

### Experimental Setup

Figure 2a shows the hysteresis frame device for the determination of the B-H hysteresis. Figure 2b shows schematically the positions of the magnetization coil and the magnetic field probe. The specimen segments for magnetic (160 mm) and microstructural investigations (40 mm) are shown in Figure 2c. The measurement setup of the hysteresis frame device for magnetic hysteresis measurements was based on a power amplifier of type KEPCO, a sensor system (based on coil and magnetic field probe), and a data acquisition and evaluation MMS Hystrometer software from Fraunhofer IZFP (Saarbrücken, Germany). The magnetization coil at the yoke generated an alternative magnetic field which was channeled into the investigated specimen. The magnetic field probe was designed to measure the forming steady magnetic field strength H directly onto the surface of the specimen to avoid unsteady magnetic field conditions. H was measured via a transversal hall sensor type SS495A. In this case, only the tangential magnetic field was recorded. The magnetic field probe was positioned around the sample to detect the voltage M_a_ of the induction. With this setup, the B-H hysteresis loops shown in the following section were collected at different induction levels achieved with magnetization parameters for M_a_ ranging between 0.3 V and 1.5 V and a fixed frequency f = 1.4 Hz.

The shorter specimen segments were examined for mechanical and structural properties using a scanning electron microscope, X-ray diffractometer, as well as a hardness measurement instrument. The analyses of the retained austenite (RA) content, as well as the residual stresses, were carried out with an X-ray diffractometer PANalytical X’Pert PRO MRD, shown in Figure 2d, using Cu-Kα_1_-radiation at 40 kV tube voltage and a 40 mA tube current. Since a rather homogeneous distribution of the microstructural properties was to be expected for all investigated specimens, all investigations focused on the bulk material. Hence, XRD measurements were taken at the center of each cross-section, as shown in Figure 2e, with the spot size changing between 2.4 × 5 mm for an incidence beam angle of ω = 20° and 2.4 × 2.2 mm for ω = 50° when analyzing the RA content. Residual stresses were measured at the same position in horizontal and vertical directions perpendicular to each other. The determined residual stresses show that for all specimens, a nearly residual stress-free condition was present after heat treatment, as the residual stresses were within +/− 50 MPa. Hence, in the following analyses of the influence of the material state on the magnetic properties, residual stresses were considered to be negligible. The cross-section shown in Figure 2e was obtained by cutting the 40 mm segment at about a quarter of its length and then electrolytically polishing it. Thus, the undesirable effects of cutting, i.e., the possible influence on the phase composition or residual stresses was eliminated [20]. The Rietveld method [21,22] was applied to quantify the RA content by using PANalytical’s HighScore Plus 3.0 software (Almelo, Netherland). The residual stresses of the first order were determined using the sin²-ψ method and PANalytical’s X’Pert Stress 2.0 (Almelo, Netherland) software applied to the (211) martensite lattice plane since it was predominantly present in all material states.

The microstructural images were obtained by using an SE detector of a scanning electron microscope (SEM), type FEI Quanta 600. An acceleration voltage of 20 kV and a working distance of 12 mm was used. For visualizing the depicted microstructures, the specimen cross-sections, presented in Figure 2c, were wet ground, mechanically polished, and subsequently subjected to Nital etching prior to SEM imaging. The etchant used was two percent alcoholic nitric acid.

To characterize the mechanical properties, Vickers hardness measurements (HV 30) were carried out on the cross-section of the 40 mm segment to compensate for local inhomogeneities due to the greater penetration depth. The hardness measurements were carried out with a Zwick ZHU250 top-line hardness testing instrument. In each case, three indentations were performed along the dashed lines as shown in Figure 2f at approx. half the specimen radius. The indentation force of the Vickers indenter was F_max_ = 294 N for a dwell time of t = 10 s.

In the following, two different approaches to characterize the micromagnetic properties via established parameters, extracted from the respective magnetic hystereses, are presented. Firstly, the magnetic hystereses obtained under fixed magnetic induction level in the specimens, i.e., magnetic flux and, consequently, different magnetization parameters, are investigated. A constant magnetic flux requires the individual adjustment of the magnetization amplitude M_a_ for each individual specimen since the magnetic flux is a reaction to the applied magnetic field strength. Using this method, an explicit comparison of the physically established micromagnetic characteristics of the hysteresis is possible. Secondly, the magnetization parameters, i.e., the magnetization amplitude and magnetization frequency, of the hysteresis frame device are kept constant for all material states. These parameters were previously determined empirically to make sure all material states are included within the system-specific limits. This allows a more application-oriented comparison of the results with other, similar micromagnetic measurement systems, where these magnetization parameters can be selected. Furthermore, the micromagnetic material properties are compared with the mechanical or microstructural properties, and the correlations are discussed.

## 3. Results

### 3.1. Micromagnetic Characterization at Fixed Level of Induction

**Figure 3:**
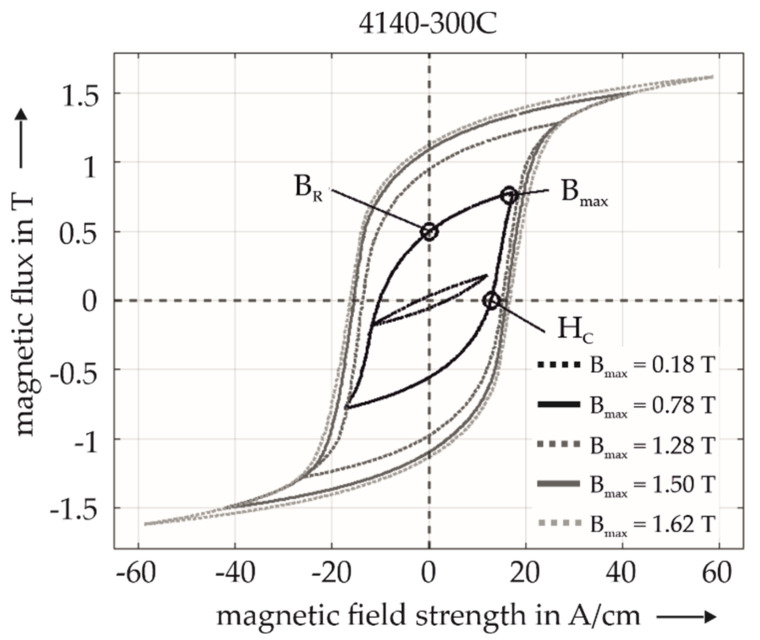
Magnetic hystereses of 4140-300C at different operating points.

The hysteresis shows the irreversible, nonlinear response of a ferromagnetic material to an external magnetization, i.e., its magnetic induction B as a function of the strength of the magnetic field H. The magnetization processes are related to domain-wall motion and magnetization rotation in magnetic domains. In a magnetically saturated material (saturation point, B_max_), the magnetic field strength is reduced slowly to zero field strength (H = 0). Then a residual magnetic induction, i.e., the remanence B_R_, remains in the material. This residual magnetization can be eliminated by applying an oppositely directed magnetic field, i.e., a negative magnetic field strength H. The field strength H at which the flux reaches the value B = 0 T is called magnetic coercivity H_C_ (here, H = −H_C_). When the strength of the oppositely directed field H is further increased, the material enters magnetic saturation with reversed polarity, i.e., B = −B_max_. When the strength of the opposite field decreases to H = 0 A/cm, the hysteresis passes through the lower branch to negative remanence, B = −B_R_. Again, induction B does not reach zero before H reaches the coercivity (here, H = +H_C_). The changes in magnetic hystereses are dependent on the magnetization parameters, e.g., the magnetization amplitude (M_a_) and the magnetization frequency (f). Figure 3 shows the formation of magnetic hystereses of 4140-300C, measured using the hysteresis frame device with magnetization parameters ranging for M_a_ between 0.3 V and 1.5 V and a fixed frequency f = 1.4 Hz. These parameters resulted in maximum magnetic flux (B_max_), ranging from 0.18 T to 1.62 T. Morever, the other characteristic points Br and H_C_ are dependent on the magnetization parameters and therefore represent different operating points.

In Figure 3, the similarity between the hystereses, with a high B_max_ of 1.28 T to 1.62 T in relation to H_C_ is striking. It shows that H_C_ no longer increases even if M_a_ is further increased. It can hence be concluded that this asymptotic value of H_C_ ≈ 13 A/cm is the “correct” physical coercivity of this material. It is well known that most ferromagnetic materials are saturated at an induction B of around 2 T. In addition, it is suitable for comparing material states to work in the quasi-static regime. All hysteresis measurements shown in the following are performed with a fixed maximum magnetic flux of B_max_ = 1 T and at a constant, quasi-static magnetization frequency f = 1.4 Hz.

**Figure 4:**
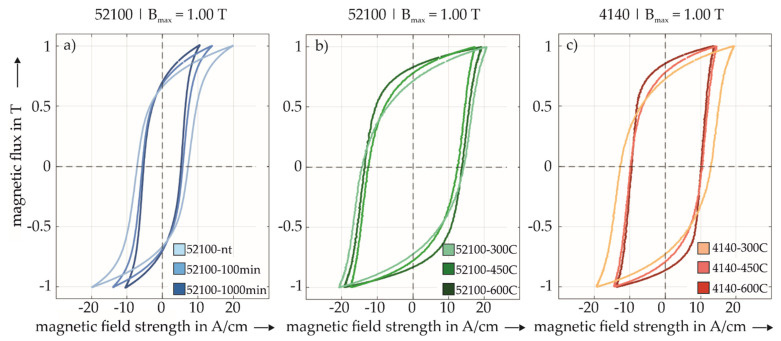
Magnetic hysteresis of the investigated specimens in different tempering states using the measurement setup shown in Figure 2a. For each hysteresis, B_max_ was set to 1 T, using various excitation amplitudes: (**a**) 52100-nt (2.1 V), 52100-100 min (1.6 V), 52100-1000 min (2.1 V), (**b**) 52100-300C (0.7 V), 52100-450C (0.6 V), 52100-600C (0.7 V), (**c**) 4140-300C (0.6 V), 4140-450C (0.6 V), 4140-600C (0.7 V).

Choosing a fixed magnetic flux amplitude allows the comparison of different material and heat treatment states and the corresponding characteristics of magnetic hystereses. For each heat treatment state, 10 measurements were taken with the same maximum magnetic flux of B_max_ at = 1 T at the same specimen position. The mean values for each annealing temperature, as well as the respective standard deviations and variances of H_C_ and B_R_, are given in Table 3. Standard deviations and variances were smaller than 1 % of the corresponding mean value which indicated good reproducibility of the measured hystereses. Figure 4 shows the magnetic hystereses of the investigated specimens at B_max_ at = 1 T in the same color scheme as introduced in Figure 1.

In Figure 4a, a comparison of the magnetic hystereses corresponding to different tempering times for AISI 52100 from non-tempered (nt) to 100 min and 1000 min are shown. A shift in the slope of hysteresis occurred, as well as the absolute permeability with B_max_ and its corresponding maximum H decreases. The 52100-nt state showed a larger magnetic hysteresis area, which resulted in higher H_C_ values. Contradictive were the 100 min and 1000 min states which were equal in terms of H_C_. Furthermore, B_R_ decreased nearly in a linear manner from 1000 min to 100 min to the nt state. In contrast, Figure 4b shows AISI 52100 at different tempering temperatures from 600C to 300C. A contraction from 600C to 450C and further widening from 450C to 300C of the hysteresis occurred. Furthermore, B_R_ increased continuously with increasing tempering temperatures from 300C to 450C to 600C. These changes in the shape of the hystereses can be easily misinterpreted, which means its shape cannot be described with only one of the two values H_C_ or B_R_, but instead both values are needed. The conspicuousness of specimen 52100-600C with respect to the RA content and its micromagnetic properties will be described in detail in Section 4. For AISI 4140 in Figure 4c, from 600C to 450C to 300C, a widening in hysteresis and therefore a slight increase in H_C_ occurred. The 300C state was therefore the most significant one, and 600C and 450C were equal in terms of H_C_. Moreover, B_R_ increased linearly from 300C to 450C to 600C and, thus, showed similar behavior to AISI 52100 in Figure 4b. The corresponding characteristic values of the hystereses are given in Appendix A.

### 3.2. Microstructural and Micromagnetic Characterization at Fixed Magnetization Amplitude and Frequency

**Figure 5:**
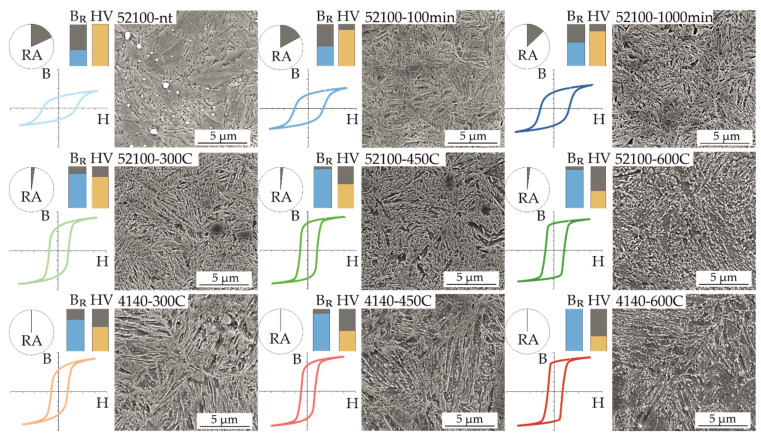
Retained austenite content RA, magnetic remanence B_R_, and hardness HV 30 in relation to the respective maximum recorded values, magnetic hysteresis in the color scheme of Figure 1, and SEM images with a 15,000× magnification of all specimens.

Due to challenging geometries of testing parts or components, micromagnetic testing is mostly done via testing heads that introduce a magnetic field from the outside surface into a small local area of the material. Because these magnetic sensors do not output the full magnetic hysteresis but instead only display individual characteristic values, e.g., B_R_ or H_C_, it is not possible to realize micromagnetic measurements for different materials or heat treatment conditions with the same value of maximum magnetic flux B_max_, as shown above. Thus, the comparison of the mentioned characteristic values was also not reliable due to different underlying hystereses. Therefore, a certain magnetization parameter set was kept constant under practical conditions. Hence, all magnetic hysteresis shown in Figure 5 were determined with the same magnetization frequency of f = 1.4 Hz and excitation amplitude of M_a_ = 1.8 V.

This illustration allows a direct, qualitative comparison of the characteristic magnetic hysteresis shapes at the same scale within and between the sets of similar material states introduced in Figure 1. The aim of this illustration is to give an overview of the microstructural, mechanical, and micromagnetic properties, showing the differences between the material states in a comparative way. It should be emphasized that despite significant differences in the material states, on the one hand, these differences could be consistently reflected in the magnetic hysteresis. On the other hand, these differences could be quantified by physics-based parameters such as remanence, coercivity, and maximum flux. With increasing tempering time at 180 °C, as well as with increasing tempering temperature and constant tempering time, comparable changes, i.e., a vertical stretching in the magnetic hysteresis shape, as well as an increase in the resulting remanence, could be observed. In the same context, the corresponding Vickers hardness HV 30 decreased significantly. In particular, among the different material states at the tempering temperature of 180 °C, a clear assignment of the tempering time to the respective retained austenite content could be discerned, which decreased with increasing tempering time. At the higher tempering temperatures and, in particular, for AISI 4140 steel, which has a lower carbon content than AISI 52100 (cf. Table 1), the retained austenite was only present to a small extent or could no longer be quantitatively detected by X-ray diffraction. These small differences in the RA content, especially at higher tempering temperatures, required a quantitative comparison of the determined phase fractions since a purely optical interpretation of the XRD spectra would be too inaccurate here. The corresponding XRD spectra are shown in Appendix B. The microstructural images of the etched material volumes shown in Figure 5 exhibit discernible differences, particularly at high tempering temperatures i.e., specimens 52100-600C and 4140-600C. The present microstructure here showed a rounding of the sharp, martensitic microstructure found in the other material states. The chromium carbides that could be identified in this context, which were present in the SEM images as both light and dark spots as a result of the preparation, were present in all states and in both materials but did not allow any direct conclusions to be drawn about the micromagnetic properties due to their inhomogeneous distribution.

**Figure 6:**
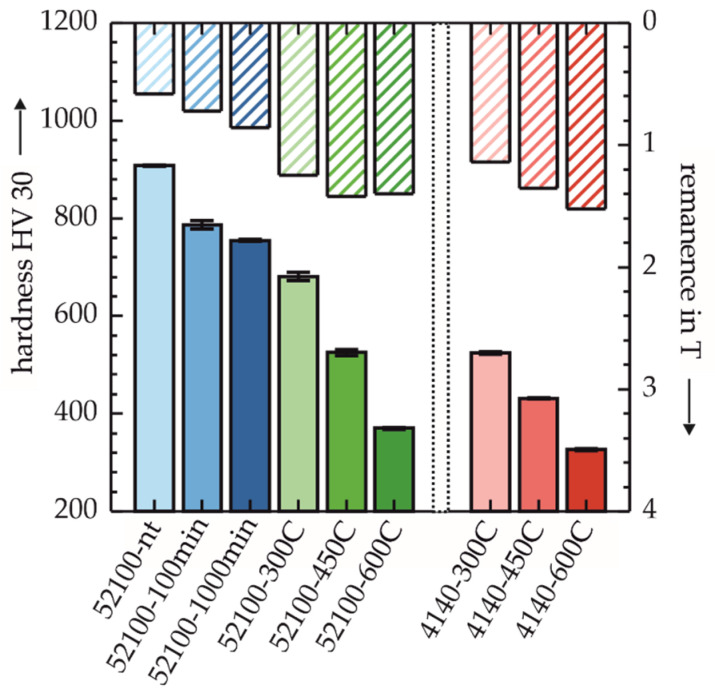
Comparison between hardness (HV 30) and remanence determined from the magnetic hysteresis.

A direct comparison of the Vickers hardness HV 30 and the remanence in Figure 6 revealed an inversely proportional relationship. The only exception to this could be seen in specimen 52100-600C. Despite the lower hardness compared to the lower tempering temperature at 52100-450C, no further increase in remanence was found. All bar graphs below separate the two materials by dashed lines for better visual differentiation. The assignments of magnetic remanence to hardness presented here thus show, in relation to the shape of the magnetic hysteresis, a stretching in the direction of higher magnetic flux and a simultaneous increase in the hysteresis slope (cf. Figure 5) as the hardness of the material decreased.

**Figure 7:**
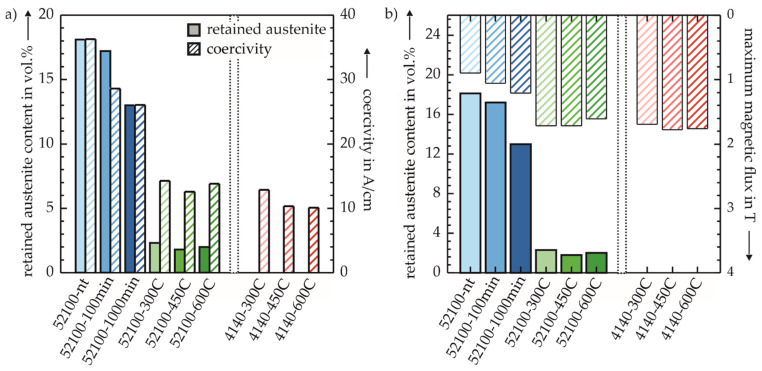
(**a**) RA content and magnetic coercivity determined at a constant magnetic flux (cf. Section 3.1.). (**b**) RA content and maximum flux determined from the magnetic hysteresis at constant M_a_ and f (cf. Section 3.2.).

Figure 7a shows that there is a very responsive correlation between the RA content and the coercivity. This was particularly noticeable with AISI 52100 and a high tempering temperature, although between 52100-450C and 52100-600C, contrary to the trend, neither the RA content nor the coercivity decreased with increasing tempering temperature. This is comparable to the phenomenon described in Figure 6 and illustrates the high sensitivity of the magneto-inductive measurement system, as both, micromagnetic and microstructural properties show an opposing trend to other material states. 

Figure 7b shows an inversely proportional relationship between the RA content and the maximum flux B_max_. Both parameters showed the largest differences between low and high tempering temperatures and thus differed qualitatively from the parameters presented in Figure 6, which also allowed clear distinctions within one of the three groups of material states (cf. Figure 1).

## 4. Discussion

The comparison of the two different investigation methods: (i) Using constant magnetic flux resp. (ii) constant magnetization parameters for characterizing the micromagnetic properties on the basis of the characteristics of the magnetic hystereses show that comparable statements can be made with both methods. Both in the characterization of the hystereses at defined magnetic flux B (cf. Section 3.1) and magnetic field strength H (cf. Section 3.2), the changes in the magnetic hystereses are characterized by the fact that with increasing tempering time of the AISI 52100 or tempering temperature for both materials, an increase in the slope of the magnetic hystereses at low field strengths can be seen. Particularly hard magnetic states, which are characterized by high magnetic coercivities H_C_ [23], are found within the respective group for all investigated specimens at low tempering temperatures or tempering times. Only specimen 52100-600C deviates from this trend in that at defined H, both B_max_ and B_R_ are lower, and at defined B, H_C_ is greater than at the lower tempering temperatures. Compared to 52100-450C, a nearly constant B_R_ can be seen with decreasing hardness, as shown in Figure 6. This contrary-to-trend lack of increase in B_R_ with increasing tempering temperature can be attributed to the following microstructural changes of 52100-600C, which can also be seen visually in Figure 5. Firstly a possible explanation for this phenomenon is the emission of carbon atoms from their forced solution of the tetragonally distorted crystal lattice of martensite and the consequent change in the morphology of the material [24]. This effect is favored by the combination of the high tempering temperature and the comparatively high carbon content of the AISI 52100 steel. In addition, this phenomenon can also be caused by the onset of grain coarsening due to recrystallization effects [24]. The effect of grain coarsening on the hysteresis would correspond to this described behavior of specimen 52100-600C, as shown in other works [25]. The discernible differences between the materials with identical heat treatment regarding the RA content (c.f. Figure 7) are mainly due to the differences in carbon content (see Table 1). After hardening under similar heat treatment parameters, the hypereutectoid steel AISI 52100 has a higher RA content than the hypoeutectoid AISI 4140. Thus, even after subsequent tempering, the slightly higher RA content in the AISI 52100 specimens can be explained [24].

In contrast to the maximum magnetic flux B_max_, the coercivity H_C_ shows a proportional relationship to the RA content. This further confirms a consistent change in the shape of the magnetic hysteresis at lower RA fractions towards significant changes in flux at comparatively small changes in field strength, provided they do not yet reach areas of saturation. Thus, clear correlations between micromagnetic and microstructural properties are detectable with both presented investigation approaches (cf. Figure 7). Furthermore, the SEM images show discernible changes in the microstructure for high tempering temperatures. It is thus shown that a wide range of different material states can be characterized reliably, in some cases even without changing the magnetization parameters. Furthermore, even minor differences in the material states are detected with all the measurement systems presented, so it can be assumed that the micromagnetic measurement system in particular has a sufficiently high sensitivity for characterizing different material states.

By using the two investigation methods presented here, it was possible to quantify the micromagnetic differences in the material states in a valid manner and, on the other hand, to achieve a versatile application of the measurement procedure and thus the transferability to comparable magnetic measuring systems. This is achieved by using fixed preselected magnetization amplitude and frequency (cf. Section 3.2). These allow the detection of the presented characteristic magnetic properties without having to use a measuring device that can map the entire magnetic hysteresis, which would allow a constant magnetic flux to be set. Furthermore, these two methods show a comparable behavior of the magnetic hystereses at different material states, which is why a reliable micromagnetic characterization of the samples can also be assumed for the method of constant magnetic field strength (cf. Section 3.2). However, it should be noted in this context that for further investigations, the magnetic hystereses should not only be quantified by H_C_, B_R_, and B_max_, among others, but also the area enclosed by the hysteresis, i.e., the magnetic hysteresis loss, should be used as a measure of the energy required to complete a full magnetization cycle [15]. Another possible approach to investigate the micromagnetic behavior would be to compare the occurring maximum slope of the magnetic hysteresis at the magnetic flux B = 0 T. As mentioned above, consistent changes in the hysteresis slope were observed here for the material states investigated. Thus, statements related to the magnetic hysteresis profile and thus additional information about the magnetization behavior at small changes of the field strength H can be made.

## 5. Conclusions

In this work, the micromagnetic parameters H_C_, B_R_, and B_max_, determined from the magnetic hystereses of specifically manufactured specimens made of AISI 52100 and AISI 4140, were compared with their mechanical and microstructural properties, respectively, and discussed. Using two different investigation approaches for micromagnetic characterization, the sample-individual material properties could be recorded reproducibly. Here, the different material states were set by quenching and tempering to defined tempering levels and tempering times. The main findings of these investigations can be summarized as follows:

- The investigated specimens can be consistently characterized in their technical and physical properties in terms of micromagnetic, X-ray, and mechanical hardness.
- The correlation of hardness to B_R_ as well as H_C_ and B_max_ to RA content shows the relationship between mechanical and micromagnetic properties, where B_R_ increases almost proportionally with decreasing hardness and B_max_ increases while H_C_ decreases with decreasing RA content.
- The use of the two different investigation methods (constant field strength or constant flux) shows a comparable characteristic behavior of the magnetic hysteresis and allows the transferability to comparable measurement systems.
- The physically-based characteristic values H_C_, B_R_, and B_max_ used for the quantification of the magnetic hysteresis profile are to be evaluated as lower limits of information for the precise description of the hysteresis profile.
- Due to the almost residual stress-free state of the specimens, the observed micromagnetic effects, in particular, can be attributed to the different RA contents as well as the discernible change in the microstructure.
- The described quantitative correlations between micromagnetic and microstructural, resp. mechanical properties of the specifically manufactured specimens allow a comparison of similar micromagnetic measuring systems since the reference ability to the respective material conditions of the specimens is ensured. This comparison will take place among multiple institutes within the context of the priority program 2086. These actions will be the first steps for a generalization and standardization of different micromagnetic measurement techniques and thus will allow an instrument-independent calibration on defined calibration specimens.

In summary, it can be stated that the presented, different material states could be reliably detected in their properties both micromagnetically and mechanically. These differences, based on the present microstructure, can thus be reliably detected close to the application by means of comparable micromagnetic measuring systems and calibrated with the help of the correlations presented, which further enables a comparison of different material states across measuring systems without additional mechanical or microstructural characterization.

## Figures and Tables

**Table 1:** Chemical composition of the investiagted batches.

Alloying Element in wt.%	C	Si	Mn	P	S	Cr	Ni	Al	N	Mo	Co	Fe
AISI 4140	0.39	0.24	0.72	0.008	0.026	1.07	0.16	0.027	0.006	0.208	0.014	96.92
AISI 52100	0.94	0.32	0.37	0.005	0.003	1.43	0.02	0.001	0.007	0.002	N/A	96.84

**Table 2:** Specimen designation and applied heat treatment.

Specimen	Material AISI	Austenitization	Quenching	Tempering
52100-nt	52100	850 °C/120 min	helium gas quenching	Non-tempered
52100-100 min	52100	850 °C/120 min		180 °C/100 min
52100-1000 min	52100	850 °C/120 min		180 °C/1000 min
52100-300C	52100	850 °C/120 min		300 °C/60 min
52100-450C	52100	850 °C/120 min		450 °C/60 min
52100-600C	52100	850 °C/120 min		600 °C/60 min
4140-300C	4140	850 °C/30 min		300 °C/60 min
4140-450C	4140	850 °C/30 min		450 °C/60 min
4140-600C	4140	850 °C/30 min		600 °C/60 min

**Table 3:** Repeatability measurement of magnetic hysteresis on AISI4140 for magnetic coercivity H_C_ and magnetic remanence B_R_. Calculation of mean value mean (…), standard deviation std (…) and variance var (…) at B_max_ = 1T.

AISI4140	Mean (H_C_)	Std (H_C_)	Var (H_C_)	Mean (B_R_)	Std (B_R_)	Var (B_R_)
300C	12.82 A/cm	0.05 A/cm	0.00 A/cm	0.73 T	0.00 T	0.00 T
450C	10.19 A/cm	0.06 A/cm	0.00 A/cm	0.79 T	0.00 T	0.00 T
600C	9.84 A/cm	0.06 A/cm	0.00 A/cm	0.86 T	0.01 T	0.00 T

**Figure A1:**
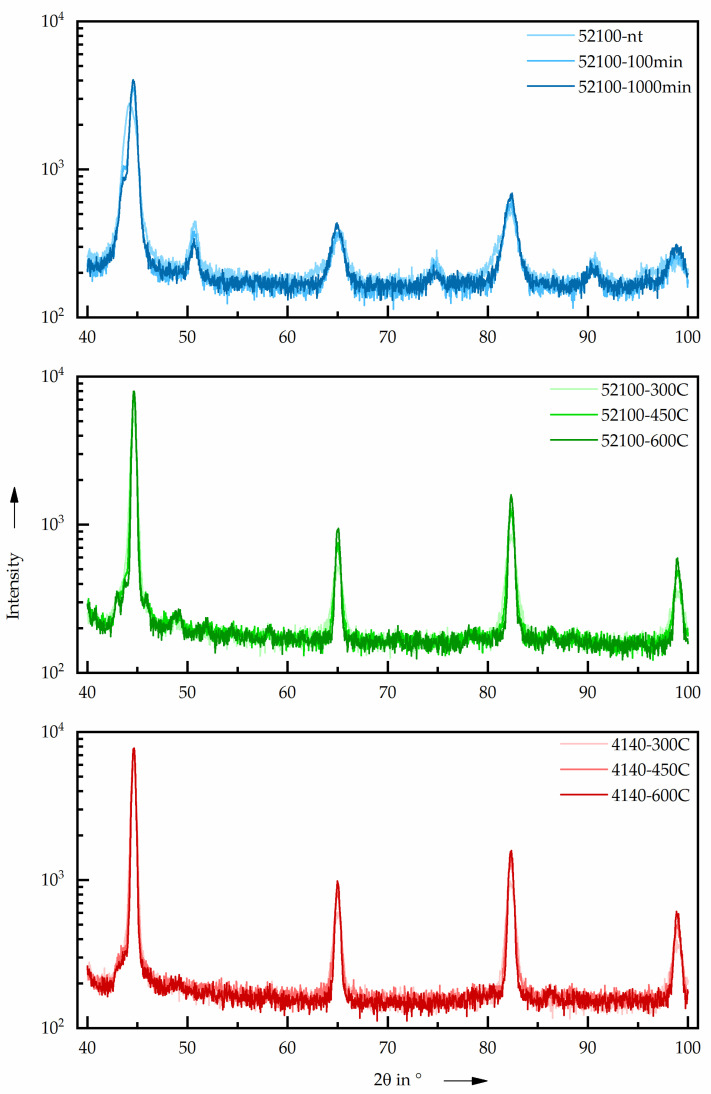
XRD diffractograms of the subjected specimens in their respective groups for better comparability.

## Data Availability

The data presented in this study are available on request from the corresponding author. The data are not publicly available due to ongoing research.

## Appendix A

Micromagnetic, microstructural and mechanical measurement data of the presented specimens in tabular listing.

**Table A1:** Short specimen segments.

Specimen	Specimen No.	RA Content in vol.%	Hardness HV 30	std. HV 30	Microhardness HV 0.1	std. HV 0.1	Mean Residual Stress in MPa	std. Residual Stress
52100-100 min	02	17.2	786.7	8.3	9734	1102	22.3	39.35
52100-1000 min	04	13	754.7	1.9	8963	198	15.3	31.7
52100-nt	06	18.1	908.0	1.4	10846	143	37.45	59.8
52100-300C	08	2.3	680.7	8.2	8060	319	−3.6	18.6
52100-450C	10	1.8	525.7	6.3	6153	195	−4.2	12.2
52100-600C	12	2	370.3	2.4	4326	363	−4.75	6.45
4140-300C	14	0	524.3	2.5	5944	131	−1.85	12.5
-	16	0.1	516.3	1.2	5964	165	17.05	15.3
4140-450C	18	0	431.3	2.1	5036	208	0.85	7.1
-	20	0	432.7	1.7	4826	100	15.8	10.8
4140-600C	22	0	326.3	2.1	3725	152	11.9	8.3
-	24	0	329.3	1.2	3766	324	7	9.55

**Table A2:** Long specimen segments.

		Data Acquired with Fixed Magnetization Parameters		Data Acquired with Fixed Maximum Flux
Specimen	Specimen No.	Remanence in T	Saturation in T	Coercivity in A/cm
52100-100 min	01	17.2	1.06	28.6
52100-1000 min	03	13	1.21	26.1
52100-nt	05	18.1	0.90	36.3
52100-300C	07	2.3	1.71	14.3
52100-450C	09	1.8	1.72	12.6
52100-600C	11	2	1.61	13.8
4140-300C	13	0	1.70	12.8
-	15	0.1	1.70	-
4140-450C	17	0	1.78	10.3
-	19	0	1.77	-
4140-600C	21	0	1.76	10.1
-	23	0	1.76	-
